# Assessment of the Potential Economic Impact of the Use of AM Technologies in the Cost Levels of Manufacturing and Stocking of Spare Part Products

**DOI:** 10.3390/ma11081429

**Published:** 2018-08-14

**Authors:** Joaquim Minguella-Canela, Sergio Morales Planas, Joan Ramon Gomà Ayats, M. Antonia de los Santos López

**Affiliations:** 1 BarcelonaTECH, Centre CIM/Departament d’Enginyeria Mecànica, Universitat Politècnica de Catalunya, Av. Diagonal, 647, 08028 Barcelona, Spain; joan.goma@upc.edu; 2 Fluidra S.A., C/Ametllers, 6, 08213 Polinyà, Barcelona, Spain; smorales@fluidra.com; 3 Departament d’Enginyeria Mecànica, Universitat Politècnica de Catalunya, Av. Diagonal, 647, 08028 Barcelona, Spain; tania.santos@upc.edu

**Keywords:** additive manufacturing, design, topological optimization, cost, stock, spare parts, free-form filament fabrication (FFF)

## Abstract

Additive manufacturing (AM) technologies are appropriate manufacturing technologies to produce low rotation products of high added value. Products in the spare parts business usually have discontinuous demand levels of reduced numbers of parts. Indeed, spare parts inventories handle myriad of products that require big immobilized investments while having an intrinsic risk of no-use (for example due to obsolescence or spoilage). Based on these issues, the present work analyses the fundamental cost factors in a real case study of a company dedicated to the supply of spare parts for fluid conduction systems. Real inventory data is assessed to determine the product taxonomy and its associated costs. A representative product of the stock is analyzed in detail on original manufacturing costs, in AM costs and then redesigned with topological optimization to reduce the AM cost levels (via design for additive manufacturing). A general equation for cost assessment is formulated. Given the specific data collected from the company, the parameters in this general equation are calculated. Finally, the general equation and the product cost reduction achieved are used to explore the potential economic impact of the use of AM technologies in the cost levels of manufacturing and stocking of spare part products.

## 1. Introduction

### 1.1. AM as an Enhancer of Potential Cost Reductions in the Context of Companies with Large Inventories

Companies providing supplies of spare parts are usually obliged to hold large sets of stocks. These inventory parts cause many issues to the companies, in the form of costs, warehouse space and logistic implications. Additive manufacturing (AM) technologies are perceived as a very powerful tool to address mass product customization, delocalized production and short series manufacturing, by maneuvering in relatively low levels of cost and short delivery periods for short series of products. This means that AM technologies could change the way that the companies supplying spare parts organize their business.

However, there is a lack of detailed studies on how the AM technologies impact the cost levels of inventories held by companies. The common case studies focus on the improvement and switch in production methods for very specific selected parts that, of course, yield major potential results. However in effect, all these studies lack of contextualization on the form of the relevance of those case studies for the overall results in the company. Issues such as (i) the representativeness of the modified parts of the overall set of products, (ii) the cost impact of the part once sold in the overall of the company performance or (iii) the importance of the part studied in the business area of the company are usually not analyzed in detail. This means that the results cannot be extrapolated from the engineering case to the business case or, in the cases it be done, that normally the extrapolation is of relatively low economic impact.

Therefore, the present work addresses the whole matter, providing an engineering case study of a relevant part applied to a relevant business area. This article assesses the technical feasibility of implementing AM technologies in the manufacturing of spare parts. In particular, the assessment is applied to a case that it is representative of the taxonomy of a relevant company for spare parts providing in the fluid conduction systems sector. It undertakes the AM production process for the initial (conventional) design and evaluates the cost levels in comparison with the conventional manufacturing technologies. Furthermore, it performs a redesign of the case study part by means of topological optimization, validating the results both by means of computer aided and physical material testing methods.

Moreover, the study case contains the study of the context characteristics and consequences of the engineering case. It enunciates a frame for cost modelling that can be extended to other companies for further comparison and analysis, it analyzes the cost factors for the parts in the inventory and it finally produces an estimation of the possible impact of the utilization of AM over the cost levels of manufacturing and stocking of spare part products.

This work does not address the design of a new inventory policy in the sense of the number of units to be held. Instead, the present work analyses the possible cost reduction while maintaining the same quantities of stock parts. In this manner, the purpose of the present study is to quantify, the potential impact of the application of AM technologies to the supply inventory parts in a relevant context in a realistic manner (not optimistic or pessimistic). The significance of the work relates to the fact that this refers to a real engineering case study with relevant data, that could be used in future references for benchmarking the state of the art as the frame and methodology can be replicable with different products and technologies.

### 1.2. Research Background and Motivations

#### 1.2.1. Additive Manufacturing: Production and Design

AM technologies, also commonly referred as 3D printing technologies, comprise numerous different techniques concerned with the materialization of three-dimensional digital models as physical objects, without the need of molds or special tooling. All these techniques have a process in common—that the material is added and consolidated layer by layer, which is the reason why many authors refer to them as additive layered manufacturing technologies. This specific manner of construction consolidation yields a specific directional material behavior effect in the direction in which the layers are added (usually taken as ‘z’ direction, and so named as ‘z-effect’), thus making the parts to adopt an anisotropic behavior being usually the ‘z’ direction the one yielding the least traction capabilities compared to the other directions ‘x’ and ‘y’.

AM technologies can certainly be classified by two different criteria, i.e., depending on the physical solution in the systems for (i) material incorporation and for (ii) energy incorporation. Usual material incorporation procedures include feeding the raw material from a point (0d), from a line (1d), of an entire layer (2d) or in a bed (3d). Usual energy incorporation procedures include applying it to the raw material in a single point (0d), within a line (1d) or within a plane (2d). Very recent approaches in holographic patterning is opening new possibilities for the application in (3d) [1]. The classification in the AM systems is crucial as it determines the materials that can be processed, the characteristics of the parts that can be obtained and the cost levels of each specific technology.

Generally speaking, AM technologies make possible to manufacture functional parts with complex design geometries, offering the possibility to optimize the mechanical properties of a part while minimizing the weight and so the energy required in their manufacturing [2]. To this regard, the topological optimization techniques deal with the material distribution within a part domain, where material density can be increased or reduced therefore changing the rigidity of each specific domain [3], and even materializing a continuous density graduation.

One particular set of AM technologies that has widespread since the expiration of many patent protections (during 2005–2010) is the fused filament fabrication technologies (FFF). In FFF, the material is incorporated in the part from an extrusion head, being this an application of both (i) and (ii) as a point (0d). FFF processing has relatively low-cost levels of hardware and materials, as well as a huge list of possible materials to be processed [4]. During the last years, industry and academia have researched and developed solutions that cover industrial needs and that are capable to address market niches, and so its use has spread and the penetration in working environments has deepened. These facts position very well the FFF technologies to be utilized as means for production in short runs of delocalized production.

The mechanical properties of the parts manufactured via FFF depend on different parameters such as the internal structure, the orientation in the construction platform and the generation of printing paths [5]. Because of its nature, parts manufactured by FFF technologies have an intrinsic danger of delamination between construction levels, which mean that the construction direction must consider the different working modes that the part will have. Because of the so-called z-effect, introduced above, FFF technologies are far from achieving isotropic parts and its mechanical properties differ from the different construction planes [6]. In the literature, this has been extensively assessed and quantified, especially in computer aided engineering and under static loading conditions. Furthermore, some studies have been conducted to assess the behavior of printed parts under dynamical loads; yielding results more adjusted to the real physical performance of the parts [7].

Moreover, the transition of conventional manufacturing technologies to AM procedures imply many added challenges in the fields of design and materials use for each specific AM technology [8]. Again, there is a very important need for the topological optimization of the parts, which is key for decreasing the costs of the parts, the environmental impact as well as the material usage. This optimization process must be addressed during the design stages of the parts. It requires a detailed study of the working conditions considered for the part and it is normally undertaken by setting an objective function with the constraints of: Material properties, geometrical characteristics, design domain, loads, and supports. The optimization itself is performed by means of computing, iterating solutions between different models. However, it is necessary to allow some human design input to conduct certain aspects of optimization, which is the reason why it is so needed to have tools that allow to ease the free-form design [9]. The overall result of the design for additive manufacturing (DfAM) methods are three-dimensional models redesigned, normally with complex structures and capable to be manufactured by a specific AM technology.

Quantifying the impact of the possible substitution of a conventional manufacturing technology for an AM technology is an open technological challenge that needs to involve both technical knowledge (product optimization) and economic analysis (costing policies). The recent times have been of a high increase in the sales of desktop manufacturing systems—the units believed to be already sold in 2017 nearly double the figures of two years earlier, reaching over 528,952 units worldwide [10], so technologies such as FFF are now more available than ever to industrial companies. Now the challenge is to explore up to which point the current technology can reach relevant product materialization.

#### 1.2.2. Cost Modelling of Additive Manufacturing Technologies

The manufacturing costs levels of manufacturing AM technologies can be characterized relating it to three different factors: (i) Part weight, (ii) part dimensions, and (iii) construction time.

Part weight (i) is a common improvement claim for AM from conventional manufacturing technologies. This is because AM technologies only consolidate the material that would be a slice of the product. The material not used can be reutilized, thus saving raw material. However, the reality is that the material consumption to be accounted is higher in some AM methods. For example, in bed technologies, such as selective laser sintering (SLS) there are limits on the number of recirculation’s of material in the bed, so further material consumption must be considered. Fruthermore, some deposition technologies, such as FFF, require printing supports (normally in the form of a honeycomb) to manufacture some of the parts, that will also be a scrap material rate.

Concerning (ii) and (iii), some industrialists prefer to treat them autonomously and some combine them in the elaboration of product’s quotations. The reason is that the main factor for the cost in the volume of the part is the ‘z’ direction of the part when set in the machine for 3D printing. Moving the 3D printer head in the ‘X’-‘Y’ plane can be very fast, but the maximum amount of time is spent when moving head and bed relatively over the ‘Z’ direction. Some authors have studied in detail the consolidation parameters, for example in selective laser melting [11], at all levels: Track, layer, and 3D object. The so-called ‘hierarchical approach’ facilitates the obtention of high-quality high-density parts. In this context, some industrialists prefer having construction time (iii) as a separate cost factor to integrate better the setting-up and post-production costs while others handle them in combination to have easier cost models.

With this rationale it is clear to see that, in AM technologies, complexity does not affect the final manufacturing cost of the part. Because of this, it is significant to undertake DfAM analysis before performing the manufacturing process, so to minimize the manufacturing cost levels.

In previous works of the authors [12], the costs configuration of the AM production technologies has been formulated in function of: Machinery costs, materials costs, energy consumption costs and labor costs. Implicit in these terms there are the factors of mass, ‘z’ dimension and construction times. Some other costs models [13] differentiate the well-structured direct production costs (labour, materials, equipment, etc.) from the ill-structured production costs (construction failures, transportation, inventory, etc.).

Having revised all this, in the present study, the Hopkinson and Dickens method [14] has been selected as the reference framework for manufacturing costs calculation. This method calculates the costs by splitting assumes that the energy consumption costs of the machines are negligible (assuming less than 1% of the final cost). Furthermore, the present work tries to broaden the work scope to the costs of manufacturing plus the costs caused by its stockage. Concerning this, some authors have prepared product lifecycle models for the spare parts sector [15], as it keeps being an active working topic.

#### 1.2.3. Cost Modelling and Issues of Holding Stock Parts

The evaluation of the inventory cost is a field widely addressed in the literature because of its many implications in the product supply chain. The economic order quantity (EOQ) is the more extended model used by the authors [16]. EOQ is a model that addresses how much product to order, taking into consideration the ordering costs and the holding costs. The ordering costs include some cost elements such as labor and other indirect office costs that enable to process the order. The holding cost includes the costs of storage, insurance, spoilage and others. The cost of capital is usually considered in the holding cost calculation [17] although some authors advocate for maintaining it as a separate cost factor.

Traditionally in the EOQ model, the holding costs are modeled in function of the average number of units per order. This term represents the stock cost [18,19]. Some authors introduce variations in this model, while others [20] consider two parts of a holding cost; i.e., one depending on the average number of units per order and another one depending of sudden increases in cost such as renting or renovating a warehouse for keeping extra units of product.

The inventory policies for controlling and maintaining optimal inventory levels study the balance between expenditure in ordering costs and in holding costs. This is the reason why it is necessary to develop and select the most effective inventory model yielding the optimum inventory framework minimizing its cost. Some authors [21] have assessed different models, such as lot for lot, EOQ, period order quantity (POQ), least unit cost, least total cost, least period cost, and the Wagner-Whitin model. After the assessment, this last model yielded the best results for the least total annual inventory cost.

In the frame of EOQ theory a cost model with arbitrary function is also developed [22]. In this case, the time-depending holding cost is introduced, in order to take into account the higher money effort for keeping fresh some perishable goods. Some other authors develop more complicated frameworks [23], incorporating the demand modelling as distribution functions [24], addressing the shortage implications in the ordering [25] and solving optimization problems using metaheuristics [18], genetic algorithms and multicriteria analysis [26].

Based on the literature review, there is a clear industrial need to be addressed in the present work, concerning the usual industrial parameters for decision taking; namely: Costs of labor, regional costs, and the time dimension. In the models reviewed, labor and regional costs are normally merged into the holding costs calculation. Therefore, there is a need to separate the accountability of such costs to compare the product costs when locating the supply chain in different geographical facilities.

#### 1.2.4. Case Studies and Implementation of AM Technologies to Industry Parts

Case study research (CSR) is a very suitable methodology for undertaking assessments in both the fields of engineering technology and production administration, which has been gaining importance over many different disciplines during the last years [27]. Compared to pure experimentation, which is probably the most common engineering approach, CSR can handle with the propositions on ‘how’ and ‘why’ of contemporary data without having to control behavioral events [28].

The study cases of application of AM technologies to product manufacturing in the recent literature have commonly addressed metal AM applications, and are characteristics of the companies that manufacture and sell AM equipment [29]. Some pure research experimentation approaches are capable of assessing in more detail the conditions and optimal parameters form parts obtention [30]. However, these very comprehensive studies focus on the technical product optimization that can be achieved, and do not assess whether the products improved have a defining impact on the company which produces them at its overall level. The reality is that, at the present time, most of the products optimized in such case studies are parts that are only product prototypes that are not in the main product core range of the firms. Alternatively, in some cases that the products are in the core activities of the companies, some parts fail to be economically relevant in the broader perspective.

The case study formulations can be prepared in single-case designs or multiple-case designs [28]. The results obtained in the engineering case are extrapolated and the impact is evaluated at the company level. Due to the nature of the case study, there has been a very comprehensive process to select the unit of analysis to make sure that the results can be extrapolated to the effect in the size of a company.

## 2. Materials and Methods

The presented case study methodology is grounded in the real circumstances of *Unistral Recambios*, a company that belongs to the *Fluidra* group, which accounted for a total price list cost of spare parts held in the inventory books over 15 M€ in 2017 (considered the year of the study). In such example, the large volume of references does not allow to treat all the cases one by one. In the present work, the decision taken has been to formulate a case study with a holistic (single unit of analysis) single-case design. The results obtained in the engineering case are extrapolated and the impact is evaluated at the company level.

Due to the nature of the case study, there has been a very comprehensive process to select the unit of analysis to make sure that the results can be extrapolated to the effect in the size of a company. This selection of the unit of analysis (segmentation and taxonomy) has been conducted in the phase of Study definition. In parallel to the study definition, there has been constructed the framework construction for costs evaluation.

The core of the study development has been performed in the engineering case, assessing a product relevant from the global set. The final phase is the analysis of the Impact of the optimization level achieved and the discussion of the possible extrapolation to the potential impact in both company and sector levels. From these, further achievable impact could be explored by the extension of the analysis to other product fractions and/or with the introduction of changes in the inventory policies of the company.

**Figure 1:**
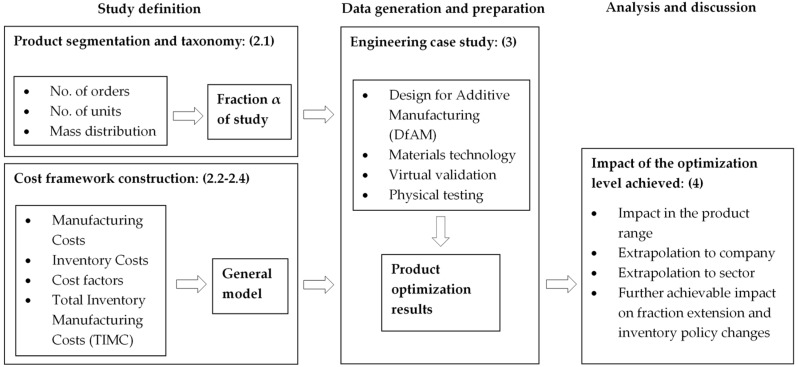
Flow diagram of the present study methodology.

The overall flow diagram of the methodology undertaken in the study is presented in Figure 1.

### 2.1. Inventory Case Study: Spare Parts for Fluid Conduction Systems

In a first exploratory analysis of the data obtained from enterprise resources planning software (ERP) utilized by the company. There are 9072 references which can be object of study, and that can be categorized as follows:

- Kits: Sets of different parts; which can be composed, for example, of a plastic part, a sealing gasket, and screws.
- Plastics: Plastic parts mainly manufacturing by injection molding, blowing or machining processes.
- Chemicals: Chemical substances such as chlorine, acids, and bases.
- Electrical: Including motors, electrical cables, and connectors, among others.
- Mechanical: Standard mechanical elements such as screws, mechanical seals, washers, etc.
- Elastomers: Comprising elements based on elastomeric materials, mainly sealing gaskets, and membranes.
- Filtration: Including filter beds, membranes, and filter cartridges.
- Measurement: Including sensors and devices for temperature measuring, alkalinity, and active oxygen, among others.
- Others: Including items purchased by external suppliers or standard plastic elements, such as junction boxes.

From this product aggregation, the category of ‘Plastics’ account for 2735 product references, that represent a 30.3% of the total number of articles considered. These are the specific targeted parts focused in the present study, as most of them could in fact be manufactured by plastic AM technologies, despite being originally designed to be manufactured by other means.

In these selected plastic product references, the next step in the methodology consists on evaluating the relative demand and rotation of each product reference. Therefore, the study analyses both the distribution of the orders and the average lot size of each of them. 

**Figure 2:**
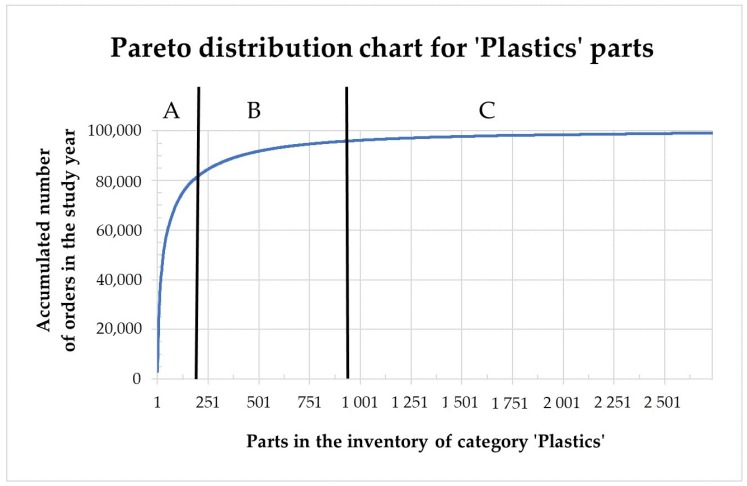
Pareto distribution A-B-C of the orders received for all references within the category ‘Plastics’. Elaborated by the authors from *Unistral Recambios* data.

Firstly, it has been studied the number of orders received for each of the references. For this reason, a Pareto-type analysis has been made showing the distribution of the number of orders of each product or reference within the category ‘Plastics’ (see Figure 2). During the study year, the 2735 product references were called in a total of 99,009 independent orders.

The Pareto analysis divides the orders received in the category ‘Plastics’ within three population levels ‘A’, ‘B’ and ‘C’. The ‘A’ sector comprises the products that represent the 80% of orders expressed in number of units. The ‘B’ sector comprises the products that represent a supplementary 15% of the orders and, finally, the ‘C’ sector includes the products that represent the 5% of the remaining orders. The specific data for this analysis is shown in Table 1. In this table it is also shown the maximum, average and minimum numbers of orders for the references classified in each sector. As a preliminary conclusion, the 166 references categorized in Sector A have continuous levels of demand, which can continue to be produced by conventional manufacturing means that lead to economies of scale. On the contrary, the references in the sectors B and C are potentially good candidates to be analyzed in the present study as they add up total of 2569 part references (93.9% of the products in the ‘Plastics’ category) with discontinuous levels of demand (29.9 orders/year in average for Sector B and 2.4 orders/year in average for sector C).

**Figure 3:**
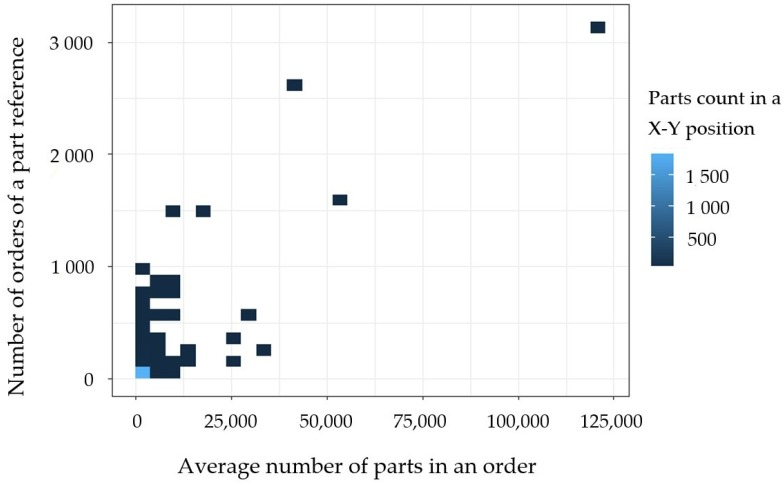
3D quantification of the rotation of the products. For each part, a plot in X and Y is configured with the number of orders received and the average number of units sold in each order within the categories ‘Plastics’. Elaborated by the authors from *Unistral Recambios* data.

Secondly, it is important to quantify the real rotation of the products; i.e., how many of each of the parts are ordered in their (possible) different orders. As stated earlier, these figures can be obtained by analyzing the average number of parts served in each of the orders for every part. Concerning to this, Figure 3 depicts the actual rotation of the products.

Figure 3 shows a very clear distribution for a company dedicated to the spare parts supply. In effect, the quantity of parts ordered in average in an independent order is relatively small. This fact, combined with what was demonstrated in Figure 2, shows how most of the parts receive relatively low numbers of orders containing relatively low numbers of units of products in each order. Only few products receive recurrent large orders. In fact, the plastic parts that do have large recurring orders are commonly small plastic bags and small foam spacers with manufacturing costs that normally fall below 0.0002 €/unit, having a limited impact in the inventory costs. Furthermore, some 142 product references accounted for zero orders in the study year.

More specific information on the distribution of the number of units in an independent order can be found in Table 2. In particular, having an average number of units in an order of 78.9 parts, in fact 75% of the orders are of 15 parts or less. Therefore, in this sector, the real industrial interest for manufacturing solutions is to respond to the orders of small batches of products.

From the mass distribution perspective of the analyzed parts, it can be produced an analogous study which is shown in Table 3. For the 2735 references analyzed in the ‘Plastics’ category, some weighty parts increase the overall average weight of a part to 2.179 kg. However, 75% of the references weight are of 1.005 kg or less. Clearly, the majority of the parts within the category have small masses.

As a summary, the taxonomy analysis undertaken in the present exploration yields that the prototype part in the ‘Plastics’ category, for this company in this sector:

1. Received 80 or less orders in the study year, so it has a discontinuous level of demand (receives less than five orders per week),
2. The ordered quantities were very low. Being conservative, most products demand fall below 2400 of total units in the period (which would imply, for example, 80 independent orders of 30 parts of the same product), so do not justify manufacturing in long runs, and
3. The mass of each part is below 1 kg of weight, so it is sensible to be manufactured by means of AM technologies.

Therefore, the case study will focus on the parts that fulfil these conditions and will be referred as the fraction ‘α’ of the inventory.

### 2.2. Total Inventory Manufacturing Costs

In order to quantify the costs in its context, it can be formalized the total inventory manufacturing cost (TIMC), which can be calculated by making the addition for all parts in the inventory of its number and manufacturing cost, as formalized in Equation (1):

(1) $$TIMC=\sum_{i=1}^{i=n}\sum_{j=1}^{j=m}\left(Q_{i}^{j}\cdot C_{MANi}^{j}\right),$$

where:

- (*TIMC*) is the global aggregate total inventory manufacturing cost
- (*i*) is a part in the inventory
- (*j*) is an order manufactured and hold in the inventory
- ($Q_{i}^{j}$) is the number of repetitions of product ‘*i*’ in the order ‘*j*’
- ($C_{MANi}^{j}$) is the manufacturing cost of product ‘*i*’ in the order ‘*j*’

In particular, $TIMC_{\alpha }$ can be used to refer the Total Inventory Manufacturing Cost (TIMC) of a subset of products ‘α’, in particular those belonging to the filtering undertaken with the ‘Plastics’ category.

### 2.3. Costing Model for Injection Molding Parts

In the present study, the calculation of the costs for manufacturing a part utilizing injection molding technologies is performed as indicated in Equation (2):

C_IM_ = C_E_ + C_TOL_ + C_L_ + C_MAT_, (2)

where:

- (C_E_) is the equipment cost per part, calculated as a ratio of the utilization time of the equipment for the manufacturing of a part, considering the equipment depreciation, the energy average consumption and the maintenance associated
- (C_TOL_) is the tooling cost per part, calculated as a division of the total tooling costs by the total number of parts manufactured
- (C_L_) is the labor cost per part, calculated as a multiplication of the time dedicated by a worker to a part by the labor hourly cost
- (C_MAT_) is the material cost per part, calculated as a multiplication of the product mass by the material cost per weight unit

In this costing model, C_E_ and C_L_ are function of the processing time required, and C_MAT_ is function of the material weight utilized.

### 2.4. Costing Model for Additive Manufactured Parts via Fused Filament Fabrication

In the present study, the calculation of the costs for manufacturing a part utilizing AM technologies is performed as indicated in Equation (3):

C_AM_ = C_E_ + C_L_ + C_MAT_, (3)

where:

- (C_E_) is the equipment cost per part, calculated as a ratio of the utilization time of the equipment for the manufacturing of a part, considering the equipment depreciation and the maintenance associated cost. In AM, the product physical dimensions (volume) affects the processing time; so, it is implicit in this cost factor. The energy average consumption is neglected following the model in Section 2.
- (C_L_) is the labor cost per part, calculated as a multiplication of the time dedicated by a worker to a part by the labor hourly cost
- (C_MAT_) is the material cost per part, calculated as a multiplication of the product mass by the material cost per weight unit

In this costing model, again, C_E_ and C_L_ are function of the processing time required, and C_MAT_ is function of the material weight utilized.

### 2.5. Costing Model for Parts in Stock

In the present study, the calculation of the costs generated for a stocked part is make as indicated in Equation (4):

Cs = C_MAN_ + C_TRA_ + C_H_ + C_MGT_, (4)

where:

- (C_MAN_) is the cost of manufacturing of a part, calculated as in previous sections, depending on the manufacturing technology utilized
- (C_TRA_) is the cost of the transportation incurred when moving the part from the production unit to the warehousing facility
- (C_H_) is the cost of holding stocked the part, considering the physical space cost in the warehouse and other derived costs such as the insurance cost for the part
- (C_MGT_) is the cost of managing the stocked part, considering the labor in warehousing and surveillance and other derived costs such as the software license costs for the part

In this costing model, C_MAN_, C_TRA_, C_H_ and C_MGT_ are function of the processing time required, of the material weight and of the physical dimensions of the part. For this reason, Cs can be expressed as shown in Equation (5):

(5) $$\begin{array}{r} \mathrm{Cs}=\left(K_{MAN}^{t}\cdot t_{MAN}^{}+K_{MAN}^{m}\cdot m+K_{MAN}^{v}\cdot v\right)+(K_{TRA}^{t}\cdot t_{TRA}^{}+K_{TRA}^{m}\cdot m+K_{TRA}^{v}\cdot v)\;\;\;\\ +\left(K_{H}^{t}\cdot t_{H}^{}+\cdot K_{H}^{m}\cdot m+K_{H}^{v}\cdot v\right)+\left(K_{MGT}^{t}\cdot t_{MGT}^{}+\cdot K_{MGT}^{m}\cdot m+K_{MGT}^{v}\cdot v\right) \end{array}$$

where:

- (*K_i_*) are the constants associated to the different cost factors
- (*t_i_*) are the processing times for each part of the process (manufacturing, transportation, holding and management
- (*v*) is the envelope volume for a part, calculated by multiplying the maximum dimensions in the three cartesian directions (X·Y·Z).

This cost modelling framework is interesting for global companies as it enables a comparison of different manufacturing and inventory costs incurred in different locations, as far as the labor costs, the land costs and the logistic chains are quantified; as well as the processing times required, material weight and physical dimensions of the parts.

If a designer can reduce the mass and the manufacturing processing times of a product, without modifying any of the rest of factors, the variation of Cs for a single part in the stock can be calculated as shown in Equation (6):

(6) $$\Delta \mathrm{Cs}=\left(K_{MAN}^{t}\cdot \Delta t_{MAN}^{}+K_{MAN}^{m}\cdot \Delta m+K_{TRA}^{m}\cdot \Delta m+K_{H}^{m}\cdot \Delta m+K_{MGT}^{m}\cdot \Delta m\right),$$

where:

(7) $$\Delta t_{MAN}^{}=t_{MAN}^{Conventional\;process}+t_{MAN}^{DfAM\;process},$$

(8) $$\Delta m=m_{}^{Conventional\;product}+m_{}^{DfAM\;product},$$

Or, grouping the terms of Equation (7), as shown in Equation (10):

(9) $$\Delta \mathrm{Cs}=\left(K_{MAN}^{t}\cdot \Delta t_{MAN}^{}+K_{MAN}^{m}\cdot \Delta m\right)+\left(K_{TRA}^{m}+K_{H}^{m}+K_{MGT}^{m}\right)\cdot \Delta m$$

Which, in the aggregation of all the changes in Cs for all the parts in the stock, can be calculated as expressed in Equation (11):

(10) $$\sum_{i=1}^{i=n}\Delta {\mathrm{Cs}}_{i}=\;\sum_{i=1}^{i=n}\;\Delta C_{MANi}+\sum_{i=1}^{i=n}\left(K_{TRA}^{m}+K_{H}^{m}+K_{MGT}^{m}\right)\cdot \Delta m_{i}.$$

Or, in terms of the TIMC, as expressed in Equation (12):

(11) $$\sum_{i=1}^{i=n}\;\Delta {\mathrm{Cs}}_{i}=(TIMC_{\alpha }^{Conv.\;proc.}\cdot \delta )+\left(K_{TRA}^{m}+K_{H}^{m}+K_{MGT}^{m}\right)\cdot m_{total\;\alpha }^{Conv.\;proc.}\cdot \gamma .$$

where:

- (α) is the target group for the switch to AM processes; i.e., ‘Plastics’
- (δ) is the average cost reduction in manufacturing of the parts achieved for the target group (α)
- ($m_{total\;\alpha }^{Conv.\;proc.}$) is the original total mass of the target group for the switch to AM processes; i.e.,: ‘Plastics’
- (γ) is the average mass reduction in the parts achieved for the target group (α)

## 3. Results

The product object of the case study in the present work is a real case articulated in the fluid handling industry. To be consistent in the analysis and following to the product segmentation undertaken in Section 2.1, it has been chosen a relatively common product (weighting less than 1 kg), demanded on a relatively low number of orders (less than 42 orders) and in relatively short series (less than 2400 units per order in average). The product is redesigned, and the costs are assessed in the conventional manufacturing process and in an AM technology (FFF) both for the original part design and for its redesign. The reduction in the manufacturing costs are then used to infer the reduction of overall inventory costs that could be achieved through the introduction of AM.

### 3.1. Case Study (Spare Part for Fluid Handling): Product Definition

#### 3.1.1. Product Specifications

**Figure 4:**
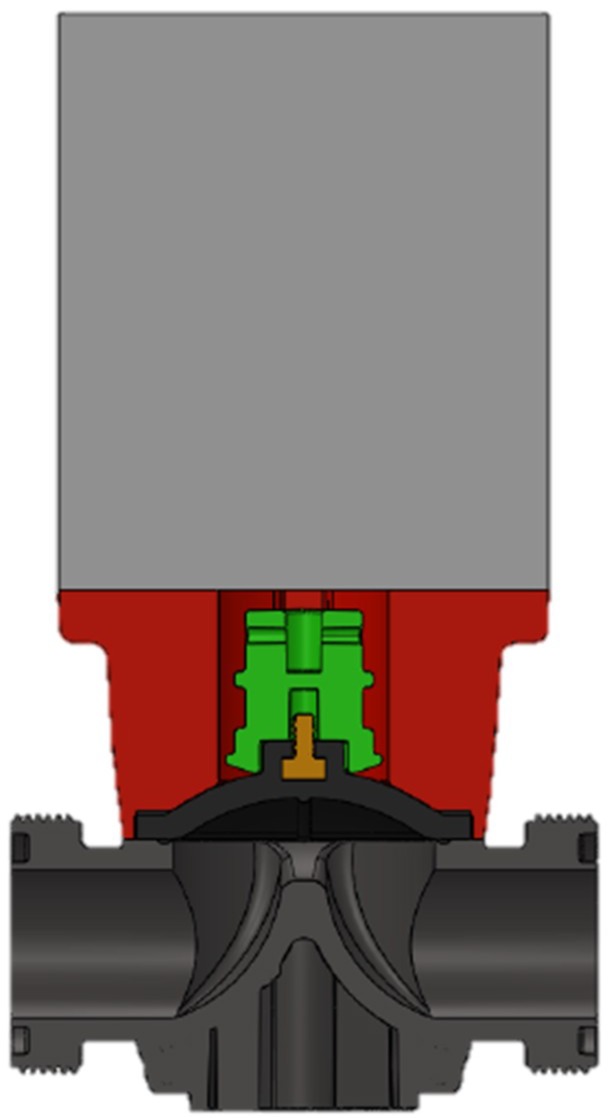
Automatic valve section, in red the part to be redesigned and assessed (support).

The overall product is a flow regulation system for fluids (an automatic valve), which contain many different parts. A pneumatic piston acts on the actuator (green) which is connected to the membrane (black) by means of a metal insert in form of a pin. The support (red) is responsible for aligning the actuator with the membrane so that it can restrict the flow and separate the pneumatic system from the hydraulic system. Finally, the body of the valve (dark gray) is responsible for the conduction of the fluid (see Figure 4). The system is fixed with stainless steel screws that go through all the parts until reaching the pneumatic piston.

In this case, the part to be studied is the support of the automatic valve. The need is to manufacture 2000 units of supports to fully supply the customer.

The technical specifications of the support require the use of materials with good mechanical and thermal properties. For this reason, technical polyamide (PA666) also used in the automotive industry, is the material selected for manufacturing the part. It is a strong, ductile and easy to print material. The filament for the present study (*Novamid^®^ ID1070*), was supplied by *Nexeo Solutions*, suppliers of the material used.

#### 3.1.2. Original Product Costs (Molding)

The case study for this inventory part starts with the costs assessment considering that this part was originally designed to be manufactured by injection molding technologies. All the economic treatment is handled in Euros as the company in the case study has its headquarters in the Barcelona region (Spain).

As it can be seen in Table 4, the cost analysis reaches a minimum total cost per part of 1.71 €, in the case of a total production volume of 275,940 parts per year over a 10 years’ production period. In the case of manufacturing only the 2000 units to supply the demand in the considered order, the total cost per part is of 21.61 €.

#### 3.1.3. Original Product Costs (AM)

As a starting point approximation, the 3D printing costs of the case study part (support) are analyzed without carrying out any type of structural redesign. The printer used for the present study is an FFF machine from the manufacturer “BCN3D” (Sigma model) that has the impact on the cost of the part accounted in Table 5. With this calculation method, the total cost per part when manufactured via AM technologies is of 40.06 € with independency of the number of units to be manufactured.

The operation data presented in Table 5 has been calculated with the BCN3D CURA software, including the number of parts per platform in the present case-2-, and the platform build time—143 h. CURA also calculates the amount of material used in the construction, given a determined level of infill; which in the present case accounts for 862 g per part.

The relevant printing parameters utilized by the BCN3D CURA software (Version 2.0, BCN3DTechnologies, Barcelona, Spain) to calculate the cited output, which will be needed for the cost calculations are the following:

○ Nozzle diameter: 0.4 mm
○ Diameter of the filament: 2.85 mm
○ Print speed: 80 mm/s
○ Layer height: 0.1 mm
○ Wall thickness: 0.8 mm
○ Platform adhesion: Brim
○ Extrusion temperature: 230 °C
○ Temperature of the hot bed: 100 °C

**Figure 5:**
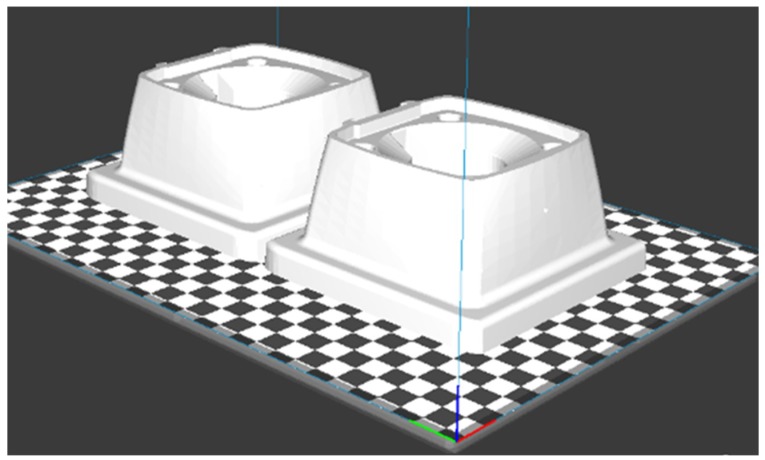
BCN3D CURA software platform simulation the case study part (support) in the original design model.

BCN3D CURA software graphical interface is capable of representing the parts in 3D located in the construction platform as they will be obtained once the parts manufacturing is completed. For the manufacturing of the case study part, it is chosen to place the parts laying on its bigger flat surface. The BCN3D CURA graphical simulation is shown in Figure 5.

### 3.2. Case Study (Spare Part for Fluid Handling): Product Redesign and Virtual Validation

#### 3.2.1. Preliminary Infill Study

As a starting point in the product redesign it is interesting to evaluate the effect of the reduction of the infill percentage of the part. Providing that the external features of the case study part remain the same, the same model can be printed with a different internal structure (filler or infill). The infill modification as a percentage is a very powerful approach, due to its easiness, to reduce the amount of material needed while maintaining the necessary stress resistance of the parts. In many cases, the filling of the part does not need to be 75% to 100%. A 25% fill can also provide enough strength and saves time and print material.

Also, in FFF, very solid parts can yield to warping and deformations due to the cooling process of the part. This deformation or warping can be reduced either through good adhesion to the construction platform or also through the printing configuration. In particular, it can be significantly reduced if a less dense structure is used.

**Figure 6:**
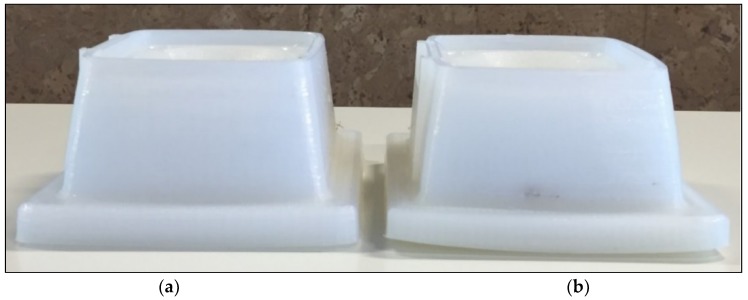
Case study printed parts: (**a**) Support manufactured with an infill percentage of 25%; (**b**) support manufactured with an infill percentage of 75%.

One of the best approaches for tuning the infill level is to test it live by printing a part. Figure 6 shows a couple of 3D printed repetitions of the case study part. The part in the left was manufactured with an infill level of 25%, while the one in the right was manufactured with an infill level of 75%. Only with this infill percentage reduction, a significant change in the shape of the part the material is reached. In particular, for the printed tests, the shape distortion is reduced between 5 and 7 mm in the four lower edges of the part. Furthermore, a significant cost reduction is observed as the printing time is reduced by a 30%.

These exploratory results can be utilized as preliminary quantitative figures for a starting point of the specific product improvement. Based on these, the product redesign, in form of DfAM, should be explored in detail undertaking the complete topological optimization and analysis.

#### 3.2.2. Product Redesign and Virtual Validation

**Figure 7:**
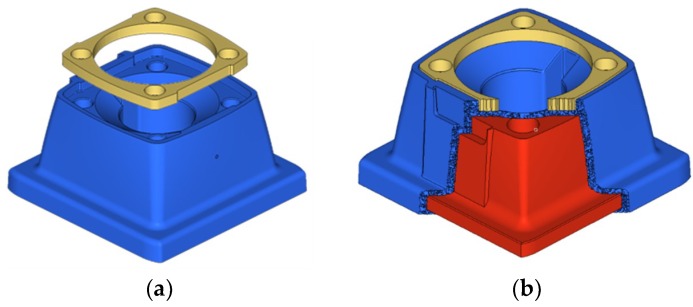
Case study part: Support (in blue) and membrane (in gold). (**a**) Support and membrane; (**b**) design domain (red) and non-design domain (blue).

The computer aided design (CAE) analysis starts with the definition of the constraints imposed by the components in the context of the system. As such, the first element to be considered for the simulation is the gold membrane that exerts a pressure on the surroundings of the housing of the support (see Figure 7a). The central part of the membrane also exerts pressure, but only when there is water pressure inside, for this reason it is presented in the study of loads. The material of the membrane is EPDM rubber (ethylene propylene diene monomer rubber), a rubber derivative with 6 mm thickness and a modulus of elasticity of 10 MPa.

The support object of the case study in the simulation is shown in Figure 7a represented in blue color. However, for the redesign and simulation means, the support is divided into two domains. The outer shell or skin, for aesthetic reasons, should remain as it is in the original design, but the interior of the support can be modified. Therefore, as presented in Figure 7b the part is divided into the design domain (interior, in red) and the non-design domain (housing or skin, in blue) with a thickness of 2 mm.

**Figure 8:**
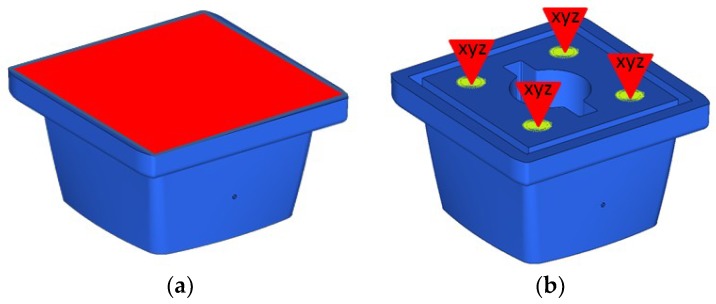
Case study part (support, in blue) and membrane (in yellow): (**a**) Surface (red) including a restriction; and (**b**) four screw fasteners support (yellow), which are the points at where the model does not allow any physical displacement over any of the cartesian directions (X-Y-Z).

Secondly, the study of the supporting surfaces of the part is addressed. The biggest surface of part is in direct contact with the base of the metallic piston and therefore, there must be included a surface restriction (see Figure 8a). As for the screws, which joins the body with the piston, it restricts movement in the X, Y and Z directions (see Figure 8b). In the center of the part there is a through housing where the rod and the actuator pass through the support until they encounter the membrane. The loads on the sides of the housing are not considered in this case study, assuming the actuator next to the stem will not contact with the walls around it.

**Figure 9:**
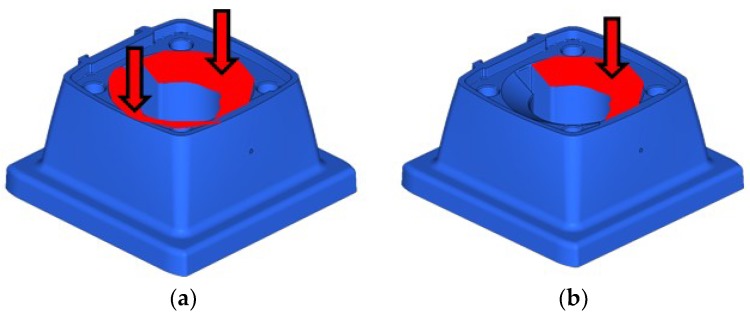
Case study part (support, in blue) with load in the conditions of: (**a**) Valve totally open; and (**b**) valve totally closed.

The third step is to identify the loads in the model and two extreme conditions have been defined: Valve totally open, and valve totally closed. In the first condition, the pneumatic actuator is resting, and the membrane passes the flow through the interior of the body at a nominal pressure of 10 bar (PN10). In this case, the pressure acts on both sides of the support (see Figure 9a). In the second condition, the pneumatic actuator applies force on the membrane stepping on the body and preventing the passage of the flow inside. In this case, the pressure acts only on one of the faces (See Figure 9b).

The body and the support are assembled by means of screws with a tightening of 6 Nm, in both cases a pressure of 1 MPa of compression has been applied on the membrane that corresponds to a deformation of 0.6 mm in its dimensions. The *Novamid^®^ ID1070* material (which is the filament used in the manufacturing of the present case study) was characterized for the two most common printing patterns (0-90 and 45-45) for the X-Y printing plane (see Table 6).

From this material characterization, the virtual testing has been executed assuming a linear simulation and utilizing the least favorable values for the material properties that are presented in Table 7 (X-Y print at 0-90). A safety factor of 30 has been considered in the voltage factor for the simulation. 

**Figure 10:**
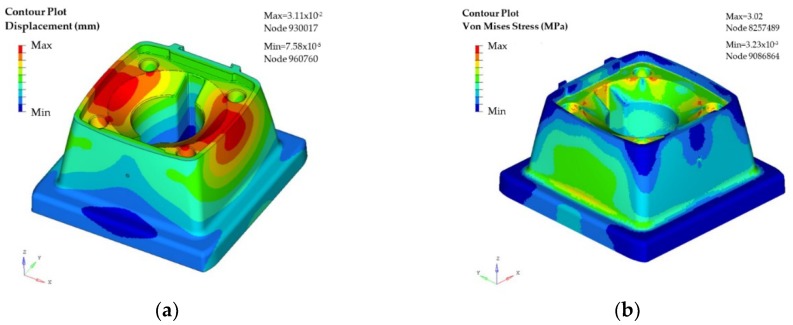
Case study part (support): (**a**) Displacements of the material for the solid model; and (**b**) Von Mises stress for the solid model.

Once the model is ready, the first simulation run is used to evaluate the behavior of the initial totally solid (100% level of infill) model of the part. With the simulation results it is possible to quantify to the material deformation (as presented in Figure 10a) as well as the Von Mises stress values (as presented in Figure 10b), providing detailed information on the different model areas. 

**Figure 11:**
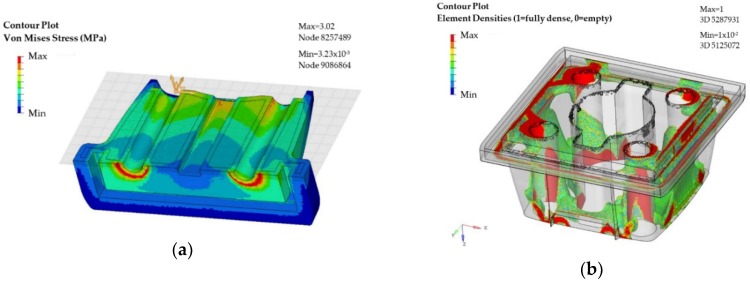
Case study part (support): (**a**) Von Mises stress focused on fixations; and (**b**) results of topological optimization.

In particular, the maximum displacement results are 0.031 mm obtained at the top of two of the sides of the part. The maximum stress of Von Mises is estimated around fixations with a value of 3.02 MPa (see Figure 10b and Figure 11a). 

These values obtained in the simulation indicate that the model is oversized and that there is room for material optimization. In this case, the objective of the topological assessment is to propose material to remove in those points where the interaction of the loads is low, and/or no displacements occur. In this manner, the objective function is set to maximize the rigidity of the model, taking into account the restrictions and supports. The results of the topological optimization are presented in Figure 11b.

**Figure 12:**
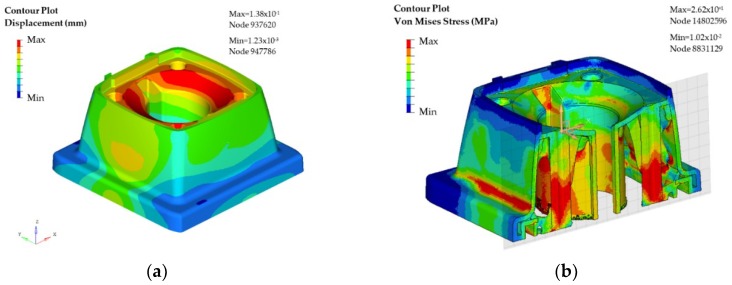
CAE of the case study part (support): (**a**) Displacement results in the optimized design, and (**b**) Von Mises stress result in the optimized design.

The results obtained in the analysis indicate the areas where it is necessary to have a greater density of material so that the part is functional according the specified mechanical requirements. Due to the asymmetry in the load results, the proposed design in this case is not completely symmetrical. However, to make sure that the part would meet the solicitations in any of the possible mounting dispositions, the denser side has been mirrored to the opposite side of the initial plane of symmetry of the part. Once the redesigned model is completed, a new simulation is run to evaluate the level of displacements and stress. The results are presented in Figure 12a,b.

In the redesigned product, the maximum displacements meet are of 0.138 mm, which are considered acceptable within the normal operation limits. Furthermore, the maximum stress (mostly due to compression efforts) is of 26.2 MPa, falling on the safe operating area.

#### 3.2.3. Manufacturing Specifications for the Product Redesign

**Figure 13:**
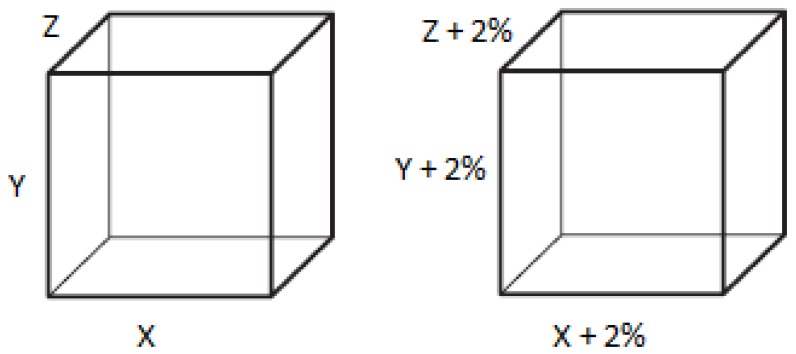
Original model (**left**). Corrected model (**right**).

To achieve the desired dimensions in the printed part, thermal contractions must be considered. For this, it is necessary to over-scale the three-dimensional model, increasing the volume in a proportion of a certain value. This value is dependent on the printing conditions, but in general, for *Novamid^®^ ID1070 1.02*, a 2% of increase in dimensions can be used. In this way, it is possible to correct the deformation contraction effect by ensuring the correct dimensions after printing (see Figure 13).

*Novamid^®^ ID1070* is printable with FFF machines whose extrusion nozzle head with temperature ranges between 220–245 °C. The most usual printing temperature is 230 °C, achieving the homogeneous melting of the material at temperatures above 225 °C. The optimum mechanical properties are observed at temperatures between 225–245 °C.

The *Novamid^®^ ID series* is a range of high performance filaments for extreme resistance and ductility. These properties are closely related to the level of crystallization of the material. In order to achieve performance similar to the standard injection molding of PA6, the high level of crystallinity in the material has been maintained. Because of this increased crystallinity, using the optimum printing conditions is necessary.

To print the new models, it was used with the BCN3D Technologies Sigma 3D with the same printing values for the parameters of nozzle diameter, filament diameter, print speed, layer height, extrusion temperature and temperature of the hot bed than in the initial printing. The infill parameters are set at 100% in this new trial of manufacturing. The reason is that once the part has undergone the topological optimization, the material allocation needs to match the computer aided design.

Concerning the adhesion of the printing part to the construction plate, the accumulation of stresses due to contractions during printing can cause the separation of the printing substrate. To this regard, adhesion can be increased by chemical bonding or mechanical bonding. To achieve a correct adhesion in the hot bed, an adhesive promoter (*Dimafix^®^*) was used, also adding an edge to increase the contact surface.

#### 3.2.4. Physical Functional Validation of the Redesigned Printed Part

After the virtual Computer aided engineering testing, the printed parts have been functionally validated in a specific test bench at the company *Fluidra*.

**Figure 14:**
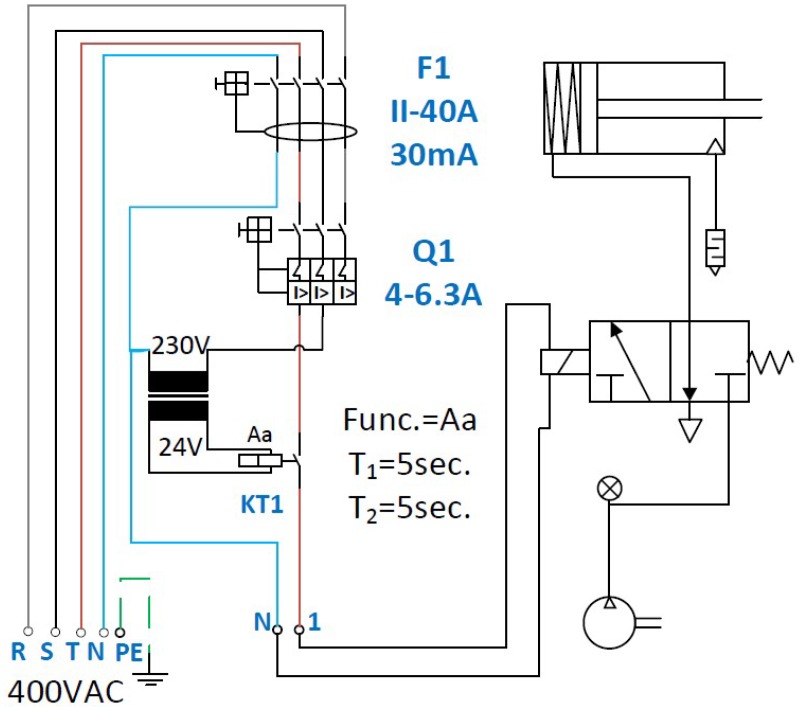
Schematics of the testing set for the pneumatic valve according to the protocol at the *Fluidra* group. Contains the solenoid valve, the piston and the compressor.

The test bench consists on a small circuit composed by a compressor that introduces pressurized atmospheric air into an accumulator that is presented in Figure 14. A pressure regulator set at 10 bar allows the air flow through a normally closed solenoid, which commands the piston that lifts the membrane. As presented previously in Section 3.1.1, the movement of the membrane (opening and closing) allows and stops the flow of liquid through the main body of the valve. Therefore, the support analyzed in the present case study is mounted between the piston and the main body of the valve. The solenoid valve is controlled by a square signal, with short cycles of five seconds with voltage and five seconds without voltage. According to the established protocol, the study part (support) must meet the same minimum operating life as the minimum membrane life, which is set at 5000 cycles.

**Figure 15:**
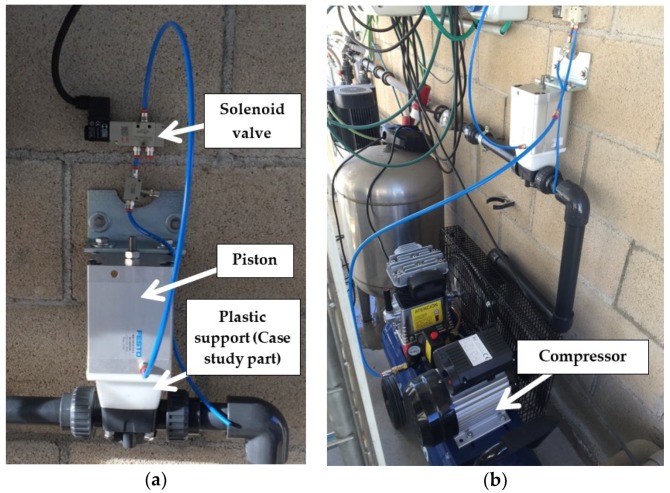
Physical testing of the printed part. It can be seen how the command circuit is mounted on a main fluid circuit. (**a**) Detailed view of the support mounted under the pneumatic piston. On top, the solenoid valve. (**b**) General overview of the fluid testing set, containing the compressor, valves, piston and the case study support.

For the validation of the present study, three printed samples were tested during 5500 cycles to check the resistance of the support in accelerated operation. All three sample parts were able to be mounted correctly, fitting in size and not having dimensional or tolerance problems. Furthermore, the three parts worked correctly showing no damage or defect in their geometry. The physical bench set is depicted in Figure 15.

## 4. Discussion

### 4.1. Cost Comparison between Additive Manufacturing Designs and Other Relevant Manufacturing Approaches

A cost study has been carried out another time with the new design to compare the results with the original design and the cost of manufacturing it by plastic injection (see Table 8).

If both designs are compared in terms of manufacturing times, a significant reduction can be observed and consequently production increases by a 41.4% per year. In addition, the reduction of the material used decreases by 72.5% compared to the original design. These factors directly impact part cost, decreasing up to 67.9%. Again, this cost is independent of the number of units manufactured. As seen before, injection molding reaches figures much lower of cost per part, providing that the number units to be manufactured is very high.

**Figure 16:**
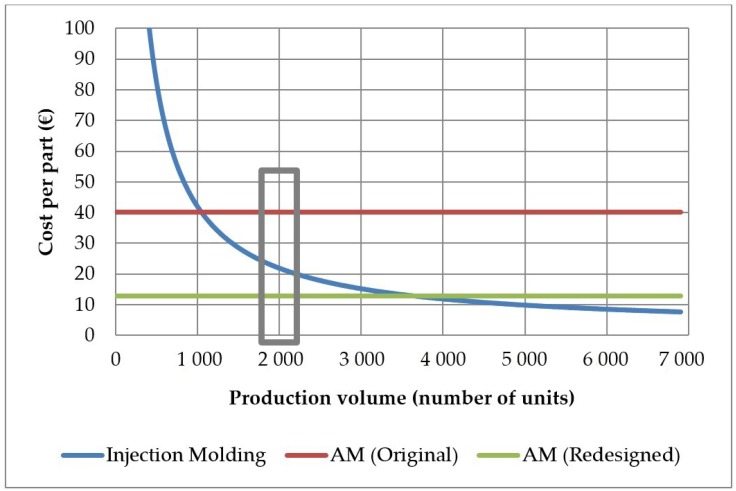
Graphical comparison of the levels of manufacturing costs of the injection molding and AM strategies.

Figure 16 summarizes the cost levels of the three production strategies. Given the order level of 2000 units, the AM redesigned geometry is the production strategy that delivers the minimum manufacturing cost (12.86 € per part). The cutting production volumes between the different production strategies yield the different decision points. In the original AM design, AM production was the best preferred option for orders of 1045 parts or below, at a cost of 40.06 € per part. Otherwise, Injection molding would be the best desired strategy. In the part redesigned for AM, AM production is the best preferred option for orders of 3605 parts or below. For larger order figures, injection molding remains as the best production strategy.

Figure 16 is thus the synthesis of the levels of cost for the current state of the art in AM and molding technologies. Providing that AM cheaper materials and faster AM machines can be developed, the horizontal cost lines for AM are expected to keep decreasing over time.

At the present time, the inventory taxonomy described in Section 2 shows that for inventory parts, normal ordering levels are very low. It has been found that it is very unlikely to require more than 2400 repetitions of a part in a year. Therefore, the zone of interest of the graph is below 2400 product units. From this selection, Figure 16 shows that substitution of manufacturing technologies—from conventional to AM—has economic sense only if DfAM is undertaken.

### 4.2. Potential Impact in the Inventory Cost

Following to the product filtering conducted in Section 2.1, the figures of the overall inventory parts considered in the present study yield a $TIMC_{\alpha }^{Conv.\;proc.}$ calculated as formalised in Equation (1) of 1,350,670 €. 

Then, in the case study, it has been possible to achieve a reduction in manufacturing costs per part of a 67.9% (δ) thanks to a reduction in the material utilised of an 72.5% in mass (γ) and a reduction on the processing time of manufacturing of a 41.4%. These percentages of reduction are aligned with the company previous experience and, according to the industrialists, can be considered a realistic average reference (not too optimistic or pessimistic).

Furthermore, given the company’s data, it is possible to calculate the factors ‘K’ needed in Equation (6) to calculate de change in costs ($\Delta {\mathrm{Cs}}_{i}$) of the products analysed. The values of the cited constants can be found in Table 9. 

With these figures and considering that the original total mass of the target group for the switch to AM processes; i.e., fraction ‘α’ of the ‘Plastics’ category ($m_{total\;\alpha }^{Conv.\;proc.}$) is of 220,514 kg, the potential impact to the inventory associated costs can be found by utilizing Equation (12) as follows:

(12) $$\begin{array}{c} \sum_{i=1}^{i=n}\;\Delta {\mathrm{Cs}}_{i}=\;TIMC_{\alpha }^{Conv.\;proc.}\cdot \left(-0.679\right)+\;\left(0.0333+0.0556+0.0222\right)\cdot m_{total\;\alpha }^{Conv.\;proc.}\cdot \\ \left(-0.725\right)=\;-934,868.56€, \end{array}$$

The overall reduction in costs is huge for the fraction ‘α’ of the product category of ‘Plastics’. Indeed, it represents a reduction of a 69.22% of its Cs. Knowing that the entire TIMC of all the product categories ascended to 6,861,642 €, the possible cost reduction associated to the stocks change in manufacturing of fraction ‘α’ of 934,868.59 €, represents a 13.62% decrease in the inventory costs. This data yields a brief estimator of what can be feasible to achieve when switching from conventional manufacturing to AM technologies in the sector of the spare parts for fluid conduction systems.

### 4.3. Considerations on Further Achievable Impact in the Inventory Cost

As some considerations on the study and its hypotheses, it is important to mention that the present work completes a case study calculating a feasible cost reduction of a part—considered representative of the group, to estimate the impact that this exercise could have when extended to a relevant share of its product range (fraction ‘α’). This estimation is performed on the context of switching from conventional manufacturing technologies—injection molding in the part analyzed—to AM technologies. Therefore, the study might be extended by selecting other parts in the inventory manufactured with different conventional technologies and so applying the methodology to yield additional cost reductions.

Also, the application of AM technologies could help achieving shorter delivery times; which could be incorporated in the study to quantify more precisely the cost reduction effects. So far, the implications that this would effectively have in the supply chain is out of the scope of the present study. Furthermore, as the batch size can be reduced to any figure, once switching to AM processes, the company might choose to modify the stocks level of the subset of products, this bringing further implications on possible savings. Moreover, some further plastic parts could be identified and redesigned from the category ‘Kits’, that could yield additional cost reductions on the overall stock cost. 

These considerations on side-effects have not been analyzed in the present study to maintain a conservative savings quantity estimation. Future research may target the study of different inventory policies for the parts of the company, starting from the costing models formulated in the present article.

## 5. Conclusions

The present study articulates and utilizes a methodology and a cost frame for assessing the potential impact of a switch in the production technologies from conventional strategies to AM strategies. The methodology consists on a taxonomy product analysis, the engineering case study analysis, the discussion of the results and the extrapolation of the findings to the potential impact in the company overall.

The taxonomy analysis is undertaken in detail to (i) select a relevant product for the case study analysis, as well as to (ii) quantify the hierarchical and economic parts catalog of the company. The engineering case study analysis covers the analysis of the product manufacturing both in the conventional process and in the AM process. The latter is evaluated in the manufacturing of the original design and of a specific product redesign implementing DfAM techniques. Being a single-case design with a single unit of analysis, the engineering case study has been screened to make sure of its validity and reliability in the context of a real engineering case from a company.

The cost frame articulated formalizes a method for calculating the impact of a product modification based on the TIMC and of several factors affecting the inventory costs, which are divided into manufacturing costs, transportation costs, holding costs, and management costs. The factors affecting the costs are dependent on basic cost generators; namely: Processing time, material weight, and material dimensions.

The engineering case study part selected is a support for an automatic valve that it is required in a volume of 2000 parts. Following the taxonomy analysis performed, it is considered a representative part of the inventory in terms of weight and demand. The original design, weighting 0.862 kg has been assessed in its costs of manufacturing. Then, topological optimization has been undertaken reaching a weight of 0.237 kg without modifying the external shape. The DfAM has been validated experimentally both in virtual and physical conditions. This enormous reduction in mass has led to cost reductions in terms of material and processing times. The estimation within the category analyzed yields that a cost reduction of 69.22% of the manufacturing costs of ‘Plastics’ parts could be achieved. When considering this impact in the company overall, it is found that a reduction of 13.69% of inventory costs could be feasible to obtain.

The methodology and the cost frame in the article are applied to a company dedicated to the provision of spare parts for fluid conduction systems. However, both methodology and cost frame could be applied to any other industrial company. The specific figure of the reduction of a 13.62% in the inventory costs can be extrapolated to other companies operating in similar contexts. In addition, analogous analysis could be done assessing different product fractions to incorporate AM technologies to other product categories. For example, it could be used in the category of ‘Kits’ for AM plastic technologies or in other categories for example with metal technologies.

Concerning the cost frame, the factors affecting the inventory costs have been specifically split into the presented division to ease the treatment of the effect of costs of the use of materials, technology, regional locations and human factor. Therefore, the same kind of study that has served to evaluate a topological optimization that has led to savings in different cost factors (volume and weight), could be undertaken with the same cost frame model for a change on the regional change in location affecting the product value chain from manufacturing to inventory.

## Figures and Tables

**Table 1:** Pareto distribution A-B-C of all references. Elaborated by the authors from thanks to the data facilitated by *Unistral Recambios* data.

References and Orders in eEach Pareto Sector	Sector A	Sector B	Sector C
Total number of references in the sector	166	513	2056
Maximum number of orders for a part in the sector	3078	80	9
Average number of orders for a part in the sector	477.5	29.9	2.4
Minimum number of orders for a part in the sector	80	10	0

**Table 2:** Distribution of the number of units demanded in each independent order.

Total Number of Orders	Max. no. of Units in an Order	Average no. of Units in an Order	Min. no. of Units in an Order	Max. no. of Units in an Order on Percentile 25	Max. no. of Units in an Order on Percentile 50	Max. no. of Units in an Order on Percentile 75
99,009	118,893	78.9	1	1	4	15

**Table 3:** Distribution of the mass in the product references.

Total Number of References	Max. Part Weight (kg)	Av. Part weight (kg)	Min. Part Weight (kg)	Part Weight on Percentile 25	Part Weight on Percentile 50	Part Weight on Percentile 75
2735	25	2.179	1.0 × 10^−12^	0.252	0.655	1.005

**Table 4:** Injection molding costs assessment for the original product at a maximum production rates and at an order level of 2000 units.

Injection Molding Manufacturing Cost Per Part		
**Operation data**		
Production rate per hour (h^−1^)	35	35
Hours per year in operation (h)	7884	7884
Production volume total (per year)	275,940	2000
**Cost of Equipment**		
Machine and ancillary equipment (€)	400,000	400,000
Equipment depreciation cost per year (€)	40,000	40,000
Machine maintenance cost per year (€)	8000	8000
Total machine cost per year (€)	48,000	48,000
Total machine cost during the time of operation (€)	48,000	347.9
**(C_E_)** Machine cost per part (€)	0.17	0.17
**Cost of Tooling**		
Mold cost (€)	40,000	40,000
**(C_T_)** Mold cost per part (€)	0.14	20
**Cost of Labor**		
Machine operator cost per hour (€)	17	17
Post-processing time per part (min)	0.5	0.5
**(C_L_)** Labor cost per part (€)	0.14	0.14
**Cost of Material**		
Material per part (kg)	0.862	0.862
Build material cost per kg (€)	1.5	1.5
**(C_MAT_)** Material cost per part (€)	1.293	1.293
**(C_IM_) Total cost per part (€)**	1.75	21.61

**Table 5:** Additive manufacturing (AM) costs assessment for the original product.

Additive Manufacturing Cost Per Part (Original Design)	
**Operation data**	
Number of parts manufactured per platform	2
Platform build time (h)	143
Hours per year in operation (h)	7884
Production volume total (per year)	110
**Cost of Equipment**	
Machine and ancillary equipment (€)	2100
Equipment depreciation cost per year (€)	262.5
Machine maintenance cost per year (€)	100
Total machine cost per year (€)	362.5
**(C_E_)** Machine cost per part (€)	3.29
**Cost of Labor**	
Machine operator cost per hour (€)	17
Set-up time to control machine (min)	5
Post-processing time per part (min)	3
**(C_L_)** Labor cost per part (€)	1.13
**Cost of Material**	
Material per part (kg)	0.862
Build material cost per kg (€)	41.35
**(C_MAT_)** Material cost per part (€)	35.64
**(C_AM_) Total cost per part (€)**	40.06

**Table 6:** Mechanical properties of *Novamid^®^ ID1070.*

Parameters		E [MPa]	ε [%]	σ [MPa]
0-90	X-Y	1,714 ± 103.83	7.21 ± 2.97	44.85 ± 2.62
45-45	X-Y	2,124 ± 80.05	14.89 ± 6.97	50.14 ± 1.86

**Table 7:** Mechanical properties imposed in the virtual testing conditions.

Parameters		E (MPa)	σ (MPa)
0–90	X–Y	1600	42

**Table 8:** AM costs assessment for the redesigned product.

Additive Manufacturing Cost Per Part (Original and Redesigned Parts)		
**Operation data**		
Number of parts manufactured per platform	2	2
Platform build time (h)	143	84
Hours per year in operation (h)	7884	7884
Production volume total (per year)	110	188
**Cost of Equipment**		
Machine and ancillary equipment (€)	2100	2100
Equipment depreciation cost per year (€)	262.5	262.5
Machine maintenance cost per year (€)	100	100
Total machine cost per year (€)	362.5	362.5
**(C_E_)** Machine cost per part (€)	3.29	1.93
**Cost of Labor**		
Machine operator cost per hour (€)	17	17
Set-up time to control machine (min)	5	5
Post-processing time per part (min)	3	3
**(C_L_)** Labor cost per part (€)	1.13	1.13
**Cost of Material**		
Material per part (kg)	0.862	0.237
Build material cost per kg (€)	41.35	41.35
**(C_MAT_)** Material cost per part (€)	35.64	9.8
**(C_AM_) Total cost per part (€)**	40.06	12.86

**Table 9:** (Ki) constants associated to the different cost factors, calculated for the 2017 inventory figures at *Unistral Recambios*.

(Ki) Constants	Values	Units
$K_{MAN}^{t}$	0.4504	€/h
$K_{MAN}^{m}$	1	€/kg
$K_{TRA}^{m}$	0.0333	€/kg
$K_{H}^{m}$	0.0556	€/kg
$K_{MGT}^{m}$	0.0222	€/kg
